# Gene model for the ortholog of *DENR* in *Drosophila grimshawi*

**DOI:** 10.64898/2026.07.14.738501

**Published:** 2026-07-16

**Authors:** Megan E. Lawson, Kylee Sanow, Mihai Fratian, Madelyn Matura, Inayah Burton, Chinmay P. Rele, Jeffrey S. Thompson, Solomon Tin Chi Chak, Kellie S. O’Rourke

**Affiliations:** 1. The University of Alabama, Tuscaloosa, AL, USA; 2. Saint Catherine University, Saint Paul, MN, USA; 3. Denison University, Granville, OH, USA; 4. SUNY Old Westbury, Old Westbury, NY, USA; 5. Carroll College, Helena, MT, USA

## Abstract

Gene model for the ortholog of *Density regulated protein* (*DENR*) in the May 2011 (Agencourt dgri_caf1/DgriCAF1) Genome Assembly (GenBank Accession: GCA_000005155.1) of *D. grimshawi*. This ortholog was characterized as part of a developing dataset to study the evolution of the Insulin/insulin-like growth factor signaling pathway (IIS) across the genus *Drosophila* using the Genomics Education Partnership gene annotation protocol for Course-based Undergraduate Research Experiences.

## Introduction

“Computational gene predictions in non-model organisms often can be improved by careful manual annotation and curation, allowing for more accurate analyses of gene and genome evolution (Mudge and Harrow 2016; Tello-Ruiz et al., 2019). The Genomics Education Partnership (thegep.org) uses web-based tools to allow undergraduates to participate in course-based research by generating manual annotations of genes in non-model species (Rele et al., 2023). These models of orthologous genes across species, such as the one presented here, then provide a reliable basis for further evolutionary genomic analyses when made available to the scientific community. The particular gene ortholog described here *Density regulated protein* (*DENR*) in *D. grimshawi* was characterized as part of a developing dataset to study the evolution of the Insulin/insulin-like growth factor signaling pathway (IIS) across the genus Drosophila.” (Myers et al., 2024).

“The IIS pathway is a highly conserved signaling pathway in animals and is central to mediating organismal responses to nutrients (Hietakangas and Cohen 2009; Grewal 2009)” (Myers et al., 2024). “*DENR* was first discovered in a human teratocarcinoma cell line because its concentration in cells increased with cell density (Deyo et al., 1998). Subsequent bioinformatic and biochemical analyses showed that the protein is conserved across eukaryotes and functions in non-canonical translation initiation (Fleischer et al., 2006; Skabkin et al., 2010). *D. melanogaster* flies homozygous for a null, knockout allele of the gene encoding *DENR* (FBgn0030802), die as pharate adults, showing a larval-like epidermis and reduced proliferation of histoblast cells (Schleich et al., 2014). Subsequent experiments using both RNAi in S2 cells and the knockout allele in larvae showed that DENR is required, along with its interacting partner MCT-1, for the proper expression regulation of a subset of transcripts required for cell cycle progression and growth. In particular, the loss of *DENR* reduces expression of the insulin receptor and makes larvae less sensitive to insulin signaling (Schleich et al., 2014), thus implicating DENR in the regulation of the insulin signaling pathway.” (Laskowski et al., 2024).

“D. grimshawi (NCBI:txid7222) is a member of the Picture Wing clade (sensu Kaneshiro et al., 1995) of the Hawaiian Drosophila. Molecular analyses place the monophyletic Hawaiian Drosophila as sister to Scaptomyza clade, which both are nested within the Drosophila subgenus of the genus Drosophila(Kambysellis et al., 1995; Baker and DeSalle 1997). The Picture Wings are so called due to their dramatically pigmented wings. D. grimshawi was first described by Oldenberg (1914), and is found in high elevation cool tropical rainforest on the Maui Complex islands where they breed on rotting vegetation (Carson et al., 1970; Carson 1983).” (Lawson et al., 2025).

We propose a gene model for the *D. grimshawi* ortholog of the *D. melanogaster Density regulated protein* (*DENR*) gene. The genomic region of the ortholog corresponds to the uncharacterized protein XP_001991685.1 (Locus ID LOC6564724) in the May 2011 (Agencourt dgri_caf1/DgriCAF1) Genome Assembly of *D. grimshawi* (GenBank Accession: GCA_000005155.1). This model is based on RNA-Seq data from *D. grimshawi* (Yang et al. 2018; SRP073087) and *DENR* in *D. melanogaster* using FlyBase release FB2024_02 (GCA_000001215.4; Gramates et al., 2022; Jenkins et al., 2022; Larkin et al., 2021).The Genomics Education Partnership maintains a mirror of the UCSC Genome Browser (Kent WJ et al., 2002; Gonzalez et al., 2021), which is available at https://gander.wustl.edu.

## Results

### Synteny

**Figure 1:**
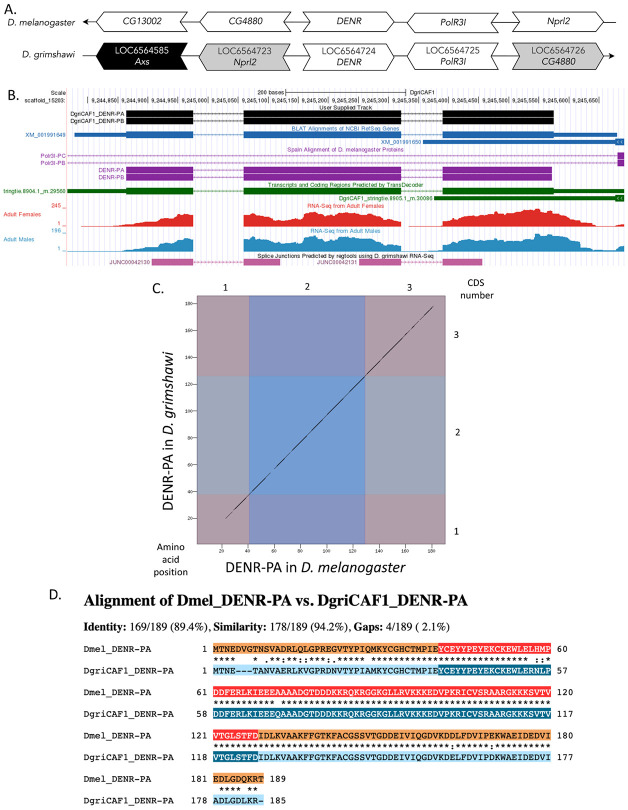
**(A) Synteny comparison of the genomic neighborhoods for *DENR* in *Drosophila melanogaster* and *D. grimshawi*.** Thin underlying arrows indicate the DNA strand within which the target gene–*DENR*–is located in *D. melanogaster* (top) and *D. grimshawi* (bottom). The thin arrow pointing to the right indicates that *DENR* is on the positive (+) strand in *D. grimshawi*, and the thin arrow pointing to the left indicates that *DENR* is on the negative (−) strand in *D. melanogaster*. The wide gene arrows pointing in the same direction as *DENR* are on the same strand relative to the thin underlying arrows, while wide gene arrows pointing in the opposite direction of *DENR* are on the opposite strand relative to the thin underlying arrows. White gene arrows in *D. grimshawi* indicate orthology to the corresponding gene in *D. melanogaster,* black gene arrows indicate non-orthology, and gray gene arrows indicate that a gene is present in both neighborhoods but in different locations relative to the target gene. Gene symbols given in the *D. grimshawi* gene arrows indicate the orthologous gene in *D. melanogaster*, while the locus identifiers are specific to *D. grimshawi*. **(B) Gene Model in GEP UCSC Track Data Hub** (Raney et al. 2014). The coding-regions of *DENR* in *D. grimshawi* are displayed in the User Supplied Track (black); coding CDSs are depicted by thick rectangles and introns by thin lines with arrows indicating the direction of transcription. Subsequent evidence tracks include BLAT Alignments of NCBI RefSeq Genes (dark blue, alignment of Ref-Seq genes for *D. grimshawi*), Spaln of D. melanogaster Proteins (purple, alignment of Ref-Seq proteins from *D. melanogaster*), Transcripts and Coding Regions Predicted by TransDecoder (dark green), RNA-Seq from Adult Females and Adult Males (red and light blue, respectively; alignment of Illumina RNA-Seq reads from *D. grimshawi*), and Splice Junctions Predicted by regtools using *D. grimshawi* RNA-Seq (Yang et al. 2018; SRP073087). Splice junctions shown have a minimum read-depth of 10 with 100-499 supporting reads indicated in pink. (C) Dot Plot of DENR-PA in *D. melanogaster* (*x*-axis) vs. the orthologous peptide in *D. grimshawi* (*y*-axis). Amino acid number is indicated along the left and bottom; CDS number is indicated along the top and right, and CDSs are also highlighted with alternating colors. Gaps in the dot plot result from decreased sequence similarity. (D) Protein alignment of DENR-PA in *D. melanogaster* (top row) vs. the orthologous peptide in *D. grimshawi* (bottom row). The alternating colored rectangles represent adjacent CDSs. The symbols in the match line denote the level of similarity between the aligned residues. An asterisk (*) indicates that the aligned residues are identical. A colon (:) indicates the aligned residues have highly similar chemical properties—roughly equivalent to scoring > 0.5 in the Gonnet PAM 250 matrix (Gonnet et al., 1992). A period (.) indicates that the aligned residues have weakly similar chemically properties—roughly equivalent to scoring > 0 and ≤ 0.5 in the Gonnet PAM 250 matrix. A space indicates a gap or mismatch when the aligned residues have a complete lack of similarity—roughly equivalent to scoring ≤ 0 in the Gonnet PAM 250 matrix. This protein alignment shows that while there is decreased sequence similarity within the beginning portion of the first CDS and the end of the last CDS of DENR-PA in *D. melanogaster* and *D. grimshawi,* there is still high conservation of the peptide sequence and the chemical properties of these regions of the peptide.

The target gene, *DENR,* occurs on chromosome X in *D. melanogaster* and is flanked upstream by *CG4880* and *CG13002* and downstream by *RNA polymerase III subunit I* (*Polr3I*) and *Nitrogen permease regulator-like 2* (*Nprl2*). The *tblastn* search of *D. melanogaster* DENR-PA (query) against the *D. grimshawi* (GenBank Accession: GCA_000005155.1) Genome Assembly (database) placed the putative ortholog of *DENR* within scaffold scaffold_15203 (CH916370.1) at locus LOC6564724 (XP_001991685.1)— with an E-value of 4e-63 and a percent identity of 52.72%. Furthermore, the putative ortholog is flanked upstream by LOC6564723 (XP_001991684.1) and LOC6564585 (XP_001991683.2), which correspond to *Nprl2* and *Axs* in *D. melanogaster* (E-value: 0.0 and 0.0; identity: 92.84% and 84.83%, respectively, as determined by *blastp*; Figure 1A, Altschul et al., 1990). The putative ortholog of *DENR* is flanked downstream by LOC6564725 (XP_001991686.1) and LOC6564726 (XP_001991687.1), which correspond to *Polr3I* and *CG4880* in *D. melanogaster* (E-value: 2e-67 and 3e-55; identity: 73.08% and 35.25%, respectively, as determined by *blastp*). The putative ortholog assignment for *DENR* in *D. grimshawi* is supported by the following evidence: synteny of the genomic neighborhood is partially conserved, and additionally while the locations of *Nprl2* and *CG4880* are not syntenic, they both remain present in the genomic neighborhood of *DENR*. All *BLAST* results used to determine orthology indicate very high-quality matches, as well.

### Protein Model

*DENR* in *D. grimshawi* has two protein-coding isoforms (DENR-PA and DENR-PB; Figure 1B). Isoforms DENR-PA and DENR-PB are identical and contain three protein-coding exons. Relative to the ortholog in *D. melanogaster*, the CDS number and protein isoform count are conserved, as *DENR-RA* and *DENR-RB* are also identical with three coding CDSs in *D. melanogaster.* The sequence of DENR-PA in *D. grimshawi* has 89.4% identity with the protein-coding isoform DENR-PA in *D. melanogaster*, as determined by *blastp* (Figure 1C). Small regions of low sequence identity were found in the first and last CDSs, but the protein alignment (Figure 1D) indicates that these regions still have very high chemical similarity across the two species. Coordinates of this curated gene model (*DENR-RA* and *DENR-RB*) are stored by NCBI at GenBank/BankIt (accession BK065241 and BK065242, respectively). These data are also archived in the CaltechDATA repository (see “Extended Data” section below).

## Methods

Detailed methods including algorithms, database versions, and citations for the complete annotation process can be found in Rele et al. (2023).

## Supplementary Material

Supplement 1

1. Zip file containing FASTA, PEP, GFF files for the gene model

## References

[R1] Altschul S. F., Gish W., Miller W., Myers E. W., & Lipman D. J. (1990). Basic local alignment search tool. Journal of Molecular Biology, 215(3), 403–410. 10.1016/S0022-2836(05)80360-2 2231712

[R2] Baker R. H., & DeSalle R. (1997). Multiple Sources of Character Information and the Phylogeny of Hawaiian Drosophilids. Systematic Biology, 46(4), 654–673. 10.1093/sysbio/46.4.654 11975337

[R3] Carson H. L. (1983). CHROMOSOMAL SEQUENCES AND INTERISLAND COLONIZATIONS IN HAWAIIAN DROSOPHILA. Genetics, 103(3), 465–482. 10.1093/genetics/103.3.465 17246115 PMC1202034

[R4] Carson H. L., Hardy D. E., Spieth H. T., & Stone W. S. (1970). The Evolutionary Biology of the Hawaiian Drosophilidae. In Hecht M. K. & Steere W. C. (Eds.), Essays in Evolution and Genetics in Honor of Theodosius Dobzhansky (pp. 437–543). Springer US. 10.1007/978-1-4615-9585-4_15

[R5] Deyo J. E., Chiao P. J., & Tainsky M. A. (1998). Drp, a Novel Protein Expressed at High Cell Density but Not During Growth Arrest. DNA and Cell Biology, 17(5), 437–447. 10.1089/dna.1998.17.437 9628587

[R6] Fleischer T. C., Weaver C. M., McAfee K. J., Jennings J. L., & Link A. J. (2006). Systematic identification and functional screens of uncharacterized proteins associated with eukaryotic ribosomal complexes. Genes & Development, 20(10), 1294–1307. 10.1101/gad.1422006 16702403 PMC1472904

[R7] Gonnet G. H., Cohen M. A., & Benner S. A. (1992). Exhaustive Matching of the Entire Protein Sequence Database. Science, 256(5062), 1443–1445. 10.1126/science.1604319 1604319

[R8] Gramates L. S., Agapite J., Attrill H., Calvi B. R., Crosby M. A., Dos Santos G., Goodman J. L., Goutte-Gattat D., Jenkins V. K., Kaufman T., Larkin A., Matthews B. B., Millburn G., Strelets V. B., the FlyBase Consortium, Perrimon N., Gelbart S. R., Agapite J., Broll K., … Lovato T. (2022). FlyBase: A guided tour of highlighted features. Genetics, 220(4), iyac035. 10.1093/genetics/iyac035 35266522 PMC8982030

[R9] Grewal S. S. (2009). Insulin/TOR signaling in growth and homeostasis: A view from the fly world. The International Journal of Biochemistry & Cell Biology, 41(5), 1006–1010. 10.1016/j.biocel.2008.10.010 18992839

[R10] Hietakangas V., & Cohen S. M. (2009). Regulation of Tissue Growth through Nutrient Sensing. Annual Review of Genetics, 43(1), 389–410. 10.1146/annurev-genet-102108-134815 19694515

[R11] Jenkins VK, Larkin A, Thurmond J. Using FlyBase, a database of Drosophila gnes and genetics. In: Dahmann C, editor. Drosophila: methods and Protocols. New York (NY): Springer; 2022. 10.1007/978-1-0716-2541-5_1 35980571

[R12] Kambysellis M. P., Ho K.-F., Craddock E. M., Piano F., Parisi M., & Cohen J. (1995). Pattern of ecological shifts in the diversification of Hawaiian Drosophila inferred from a molecular phylogeny. Current Biology, 5(10), 1129–1139. 10.1016/S0960-9822(95)00229-6 8548285

[R13] Kaneshiro KY, Gillespie RG, and Carson HL. "Chromosomes and male genitalia of Hawaiian Drosophila: tools for interpreting phylogeny and geography." Hawaiian biogeography: evolution on a hot spot archipelago (1995): 57–71

[R14] Kent W. J., Sugnet C. W., Furey T. S., Roskin K. M., Pringle T. H., Zahler A. M., & Haussler A. D. (2002). The Human Genome Browser at UCSC. Genome Research, 12(6), 996–1006. 10.1101/gr.229102 12045153 PMC186604

[R15] Larkin A., Marygold S. J., Antonazzo G., Attrill H., dos Santos G., Garapati P. V., Goodman J. L., Gramates L. S., Millburn G., Strelets V. B., Tabone C. J., Thurmond J., FlyBase Consortium, Perrimon N., Gelbart S. R., Agapite J., Broll K., Crosby M., Dos Santos G., … Lovato T. (2021). FlyBase: Updates to the Drosophila melanogaster knowledge base. Nucleic Acids Research, 49(D1), D899–D907. 10.1093/nar/gkaa1026 33219682 PMC7779046

[R16] Laskowski L. F., Burton I., Stanek T. J., Findlay G. D., Tanner S., Vincent J. A., Chak S. T., Ellison C. E., & Rele C. P. (2024). Gene model for the ortholog of DENR in Drosophila yakuba. 10.17912/micropub.biology.001017 PMC11600135 39606150

[R17] Lawson ME, Larsen CIS, McAbee M, Tanner S, Thompson JS, Stamm J, Reinke C, Rele CP, Reed LK, Gene model for the ortholog of *mts* in *Drosophila grimshawi*. 2025. microPublication Biology, submitted 10.17912/micropub.biology.000888 PMC11877148 40041767

[R18] Mudge J. M., & Harrow J. (2016). The state of play in higher eukaryote gene annotation. Nature Reviews Genetics, 17(12), 758–772. 10.1038/nrg.2016.119 PMC5876476 27773922

[R19] Myers A., Hoffmann A., Natysin M., Arsham A.M, Stamm J., Thompson J.S., Rele C.P. 2024. Gene model for the ortholog Myc in Drosophila ananassae, microPublication Biology, submitted. 10.17912/micropub.biology.000856 PMC11645546 39677519

[R20] Navarro Gonzalez J., Zweig A. S., Speir M. L., Schmelter D., Rosenbloom K. R., Raney B. J., Powell C. C., Nassar L. R., Maulding N. D., Lee C. M., Lee B. T., Hinrichs A. S., Fyfe A. C., Fernandes J. D., Diekhans M., Clawson H., Casper J., Benet-Pagès A., Barber G. P., … Kent W. J. (2021). The UCSC Genome Browser database: 2021 update. Nucleic Acids Research, 49(D1), D1046–D1057. 10.1093/nar/gkaa1070 33221922 PMC7779060

[R21] Oldenberg L. 1914. Beitrag zur Kenntnis der europäische n Drosophiliden (Dipt.). Archiv für Naturgeschichte (A) 80 (2): 1–42. [1914.05.18]

[R22] Raney B. J., Dreszer T. R., Barber G. P., Clawson H., Fujita P. A., Wang T., Nguyen N., Paten B., Zweig A. S., Karolchik D., & Kent W. J. (2014). Track data hubs enable visualization of user-defined genome-wide annotations on the UCSC Genome Browser. Bioinformatics, 30(7), 1003–1005. 10.1093/bioinformatics/btt637 24227676 PMC3967101

[R23] Rele C. P., Sandlin K. M., Leung W., & Reed L. K. (2023). Manual annotation of Drosophila genes: A Genomics Education Partnership protocol. F1000Research, 11, 1579. 10.12688/f1000research.126839.3 37854289 PMC10579860

[R24] Schleich S., Strassburger K., Janiesch P. C., Koledachkina T., Miller K. K., Haneke K., Cheng Y.-S., Küchler K., Stoecklin G., Duncan K. E., & Teleman A. A. (2014). DENR–MCT-1 promotes translation re-initiation downstream of uORFs to control tissue growth. Nature, 512(7513), 208–212. 10.1038/nature13401 25043021 PMC4134322

[R25] Skabkin M. A., Skabkina O. V., Dhote V., Komar A. A., Hellen C. U. T., & Pestova T. V. (2010). Activities of Ligatin and MCT-1/DENR in eukaryotic translation initiation and ribosomal recycling. Genes & Development, 24(16), 1787–1801. 10.1101/gad.1957510 20713520 PMC2922506

[R26] Tello-Ruiz M. K., Marco C. F., Hsu F.-M., Khangura R. S., Qiao P., Sapkota S., Stitzer M. C., Wasikowski R., Wu H., Zhan J., Chougule K., Barone L. C., Ghiban C., Muna D., Olson A. C., Wang L., Ware D., & Micklos D. A. (2019). Double triage to identify poorly annotated genes in maize: The missing link in community curation. PLOS ONE, 14(10), e0224086. 10.1371/journal.pone.0224086 31658277 PMC6816542

[R27] Yang H., Jaime M., Polihronakis M., Kanegawa K., Markow T., Kaneshiro K., & Oliver B. (2018). Re-annotation of eight Drosophila genomes. Life Science Alliance, 1(6), e201800156. 10.26508/lsa.201800156 30599046 PMC6305970

